# Analysis of Multi-Antimicrobial Resistance Patterns in U.S. Foodborne Pathogens (2015–2025) Using Data from the NCBI Pathogen Isolates Browser

**DOI:** 10.3390/pathogens15010027

**Published:** 2025-12-24

**Authors:** Daniel Lao, Leo Pan-Wang, Kenneth Tianyi Yu, Yanzhi Chen, Erin Yang, Tailin Chen, Zuyi Huang

**Affiliations:** Department of Chemical and Biological Engineering, Villanova University, Villanova, PA 19085, USA

**Keywords:** antimicrobial resistance, foodborne pathogens, *Salmonella enterica*, *Campylobacter jejuni*, *Escherichia coli*, principal component analysis, hierarchical clustering, gene–drug–pathogen interactions

## Abstract

Antimicrobial resistance (AMR) in foodborne pathogens poses a major threat to global public health and food safety. Using 9393 U.S. isolates of *Salmonella enterica*, *Campylobacter jejuni*, and *Escherichia coli*/*Shigella* collected from poultry, cattle, and swine between 2015 and 2025 and archived in the NCBI Pathogen Isolates Browser, we applied multivariate statistical analysis to characterize antimicrobial resistance patterns in isolates showing resistance to one to six antimicrobials (AMR-1 to AMR-6). Six antimicrobials—tetracycline, streptomycin, sulfisoxazole, ampicillin, nalidixic acid, and ciprofloxacin—were identified through PCA-guided clustering and frequency profiling as the principal axes of co-resistance across pathogens. Tetracycline emerged as a foundational driver of multidrug resistance, while *C. jejuni* contributed almost exclusively to single-drug resistance and *Salmonella enterica* dominated higher-order AMR categories, reflecting species-specific ecological and genomic constraints. Gene analyses revealed a progressive, modular accumulation of resistance determinants, led by efflux pumps (*mdsA*, *mdsB*), tetracycline genes (*tetA*/*B*/*O*), aminoglycoside-modifying enzymes, sulfonamide genes (*sul1*/*sul2*), quinolone resistance determinants (*gyrA*, *acrF*, *mdtM*), and β-lactamases (*bla_EC_*, *bla_OXA_*, *bla_CTX_*). Together, these results demonstrate that multidrug resistance in U.S. foodborne pathogens evolves through coordinated gene–drug–pathogen interactions rather than isolated events, underscoring the need for integrated surveillance and targeted stewardship strategies focused on the dominant antimicrobials and high-risk foodborne pathogens.

## 1. Introduction

Antimicrobial resistance (AMR) refers to the ability of microorganisms—such as bacteria, viruses, fungi, and parasites—to survive exposure to antimicrobial agents that would normally inhibit or kill them. Resistance may be intrinsic, when it is naturally present in certain species, or acquired, when it develops through chromosomal mutations or horizontal gene transfer via plasmids, integrons, and transposons. These mechanisms enable bacteria to resist treatment through enzymatic antimicrobial inactivation, target modification, reduced membrane permeability, or the activation of efflux pumps [1]. For instance, *Escherichia coli* often acquires resistance through *tetA*/*tetB* efflux pumps against tetracycline [2] or AmpC β-lactamases to resist β-lactams; 65.6% of fish isolates in the U.S. carried β-lactam resistance genes [3], while resistance to cephalosporins and carbapenems poses major clinical threats. Globally, AMR is estimated to cause 700,000 to over one million deaths annually [4,5], with severe burdens in urinary tract infections (48,700 deaths in Egypt [6]). Overuse of antimicrobials in medicine and agriculture accelerates resistance, as seen in resistance to aminoglycosides and β-lactams in Iran and Tunisia [7] and high resistance to both Extended-Spectrum β-lactamases (ESBLs) and poultry infection rates in China and Pakistan [7,8]. Under the One Health framework, which recognizes the interconnection between human, animal, and environmental health, strengthening integrated surveillance, genomic analysis, and prevention strategies targeting antimicrobial resistance is essential to protect public health, food security, and the effectiveness of modern medicine [9].

In accordance with the World Health Organization (WHO) Global priority pathogens list, *E. coli* is classified as a critical-priority pathogen, while *Salmonella enterica* and *Campylobacter jejuni* are designated as high-priority pathogens for antimicrobial resistance surveillance and control. These organisms are prevalent in both human and animal infections and often carry β-lactamases, aminoglycoside-modifying enzymes (AMEs), and plasmid-mediated resistance determinants, making them central drivers of AMR spread [8]. Intensive farming practices expose food animals to frequent antibiotic use, selecting for multi-antimicrobial resistant pathogens such as *E. coli* [8]. Surveillance studies show that over 1200 fecal samples from these animals contained widespread ESBL-producing Enterobacteriaceae [8], and resistant *E. coli* genes were detected across the same species in research conducted by the University of Minnesota [10]. Genomic analyses further underscore that their role are impacted by bovine respiratory disease (BRD), where resistance complicates treatment [11], and the Feldgarden team [12] validated AMRFinder using isolates from these species, confirming their role in disseminating clinically relevant resistance mechanisms. These may explain why *E. coli* is a leading cause of urinary tract infections and a frequent carrier of resistance to multiple antimicrobials [9]. In addition, *Salmonella* is also found to be strongly associated with foodborne outbreaks where whole-genome sequencing has linked population structure to AMR traits [13], and *Campylobacter jejuni* has developed resistance to frontline therapies such as fluoroquinolones and macrolides. Studying these pathogens thus allows comparison across species to reveal broader resistance patterns and targets the organisms most responsible for AMR infections in livestock species that dominate the human food chain and are in close contact with people [14].

Ampicillin, tetracycline, streptomycin, sulfonamides/trimethoprim, nalidixic acid, and chloramphenicol are widely used in human and veterinary medicine and are among the antimicrobials most affected by resistance in foodborne and zoonotic pathogens. These antimicrobials target first-line infections caused by *E. coli*, *Salmonella enterica*, and *Campylobacter jejuni*, yet resistance rates are alarmingly high: A 2007 study [15] reports resistance rates in poultry isolates such as tetracycline (68.5%), amoxicillin (61.4%), ceftiofur (51.3%), and spectinomycin (47.2%). Resistance mechanisms vary by class: *tet* genes driving tetracycline efflux [11,16], *gyrA* mutations leading to fluoroquinolone resistance [6], β-lactamase genes compromising ampicillin and cephalosporins [8], and plasmid-mediated quinolone resistance [17]. Streptomycin, once essential for veterinary pathogens such as *Histophilus somni*, now faces widespread resistance, while livestock surveillance shows ampicillin resistance in over 60% of *Mannheimia haemolytica* isolates [11]. The Sherry team [18] demonstrated > 99% predictive accuracy for resistance to these antimicrobials in *Salmonella* using whole genome sequencing, validating their use as core markers for multi-antimicrobial resistance (MAR) surveillance. Because these six antimicrobials are frontline therapies, heavily used in both human and animal health, and highly vulnerable to genetic resistance mechanisms, they were chosen as the focus of this study for monitoring AMR to provide the clearest picture of how resistance evolves and spreads across food animals, clinical infections, and global public health.

To systematically study multi-antimicrobial resistance in U.S. foodborne pathogens, comprehensive AMR gene data are essential, and the NCBI Pathogen Isolates Browser provides these data across food animals over time [19,20,21]. This database is a government-supported, open-access platform that compiles genomic and phenotypic data on pathogens, enabling real-time detection and surveillance of antimicrobial resistance. It provides isolate-level details such as collection date, geographic origin, resistance genes, and AMR phenotype, making it a critical tool for public health research. Studies have used it to map the distribution of key resistance determinants like *bla_CTX-M_*, *qnr*, and aminoglycoside-modifying enzymes, while also highlighting challenges in prediction accuracy [17]. The Gajdács team [9] likewise evaluated genotypic and phenotypic profiles of foodborne pathogens, while the Eckstrand team [3] applied the database’s pipelines to aquatic isolates, detecting high-risk resistance genes and uncovering overlooked reservoirs of multi-antimicrobial resistance. Related investigations have leveraged the database to identify virulence and AMR gene patterns globally [22], analyze stress response gene co-occurrence with AMR [23], and distinguish outbreak versus non-outbreak *E. coli* and *Salmonella* isolates in the U.S. [24]. More recent studies highlight its value for U.S. livestock surveillance, such as cattle [25] and poultry [26], confirming its role as a standardized data source for tracking AMR across hosts, pathogens, and antimicrobials. Two recent applications of this database indicated that three livestock-associated monophasic *Salmonella enterica* lineages frequently carried the mobile ASSuT resistance cassette [27] and that 1.84 million AMR entries, *Enterobacter* spp., showed elevated *qnrS* and β-lactam genes [28]. Collectively, these applications show that the database enables integrative, spatiotemporal AMR surveillance and provides actionable data to inform interventions against zoonotic and clinical threats.

This work builds on resources such as the NCBI Pathogen Isolates Browser but applies a new, integrative approach. Instead of examining a single pathogen–antimicrobial pair, it conducts a multi-antimicrobial, multi-species analysis of AMR by studying *Salmonella enterica*, *Campylobacter jejuni*, and *Escherichia coli* isolates from livestock hosts, including pigs, cattle, chickens, and turkeys. Resistance is evaluated across six critical antimicrobials that represent frontline therapies in both human and veterinary medicine, where high levels of resistance have been documented and where surveillance is most urgently needed. By combining phenotypic susceptibility data with genomic resistance signatures and applying multivariate statistical methods such as principal component analysis (PCA) [29,30,31,32] and hierarchical clustering [33,34,35], the study identifies resistance clusters that co-occur, reveals how they vary across pathogens. Focusing on multiple antimicrobials rather than single-antimicrobial outcomes captures the true complexity of AMR and highlights cross-class resistance patterns that shape clinical outcomes. Although centered on six key antimicrobials, the methodology provides a scalable framework that can be extended to additional antimicrobials for broader surveillance and intervention. The objective of this study is to characterize cross-species AMR patterns and co-occurring resistance clusters across these pathogens and antimicrobials, thereby providing a comparative framework to strengthen U.S. livestock-based AMR surveillance.

## 2. Materials and Methods

### 2.1. Antimicrobial Resistance Data Preparation

In this study, datasets were downloaded on 15 June 2025, from the NCBI Pathogen Detection Isolates Browser in .csv format, filtered to include only U.S. isolates collected between 2015 and 2025 with available metadata on organisms, host sources, geographic origins, and antimicrobial susceptibility test (AST) results. The 2015–2025 range was selected because it contains the largest volume of submissions and therefore best captures the most recent AMR trends. The final dataset contained 9393 isolates and 215 variables, represented as 215 columns encompassing metadata fields (e.g., organism, host source, location, and collection date). It includes three key foodborne pathogens (*Salmonella enterica*, *Campylobacter jejuni*, and *Escherichia coli*) sampled from four major livestock hosts (chicken, turkey, beef, and pork). The dataset forms a 9393 × 215 matrix, as shown in Table 1, with Columns 1–4 corresponding to metadata, Columns 5–52 capturing antimicrobial susceptibility profiles for 48 drugs (binary coded as resistant = 1, susceptible = 0), and Columns 53–215 containing 163 AMR genes identified by NCBI’s AMRFinderPlus (Version v4.0.23), coded as binary indicators of gene presence or absence. Although resistance information was available for 48 antimicrobials, six were prioritized using principal component analysis (PCA) and clustering (details provided in the next subsection) for investigation due to their clinical relevance and frequency in foodborne pathogens: ampicillin, ciprofloxacin, nalidixic acid, sulfisoxazole, streptomycin, and tetracycline. The samples were then grouped into categories based on the number of antimicrobials to which resistance was detected; for example, Data_AMR_4 contains isolates resistant to four antimicrobials. These structured data frames (e.g., Data_AMR_4, Data_AMR_5) were imported into RStudio (Version 2025.09.2+418) for downstream analysis of resistance patterns.

### 2.2. Methods

**Figure 1:**
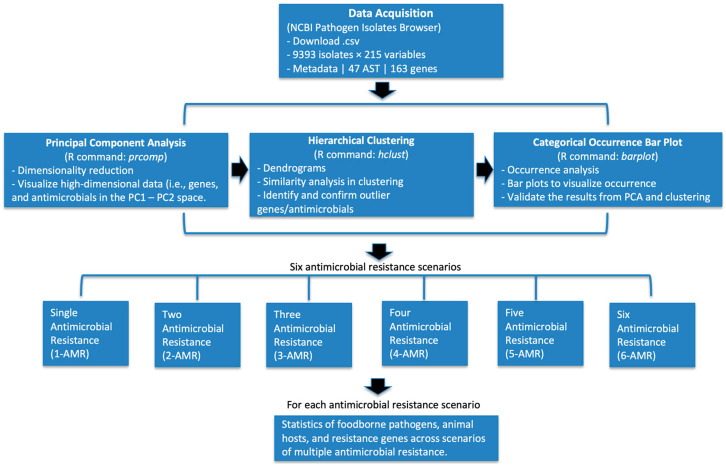
Overall workflow of antimicrobial resistance (AMR) analysis. Data were obtained from the NCBI Pathogen Detection Isolates Browser (2015–2025, U.S. isolates) and processed for antimicrobial susceptibility testing (AST) and resistance gene profiles. Principal component analysis (PCA) and hierarchical clustering were applied to identify and validate outlier antimicrobials. Categorical occurrence bar plots were used to visualize and confirm resistance distributions. Based on these results, isolates were grouped into six antimicrobial resistance (AMR) scenarios, ranging from single (1-AMR) to six (6-AMR) antimicrobial resistance. For each AMR scenario, a complementary analysis was performed for statistical summaries of pathogens and AMR genes.

The outline of the data analysis methods used in this study is shown in Figure 1. Data were downloaded from the NCBI Pathogen Detection Isolates Browser in .csv format, filtered for U.S. isolates from 2015 to 2025 with complete metadata on organisms, hosts, geographic origins, and antimicrobial susceptibility testing (AST). The datasets were processed into matrices, and resistance genes with very low appearances or variance were removed to reduce noise. In particular, a 1% frequency threshold was applied to focus the analysis on resistance genes that are more commonly observed across isolates and therefore most relevant for identifying major antimicrobial resistance patterns and clinically meaningful co-resistance trends. Principal component analysis (PCA) and hierarchical clustering were then applied to the resistance profiles, enabling the selection of six priority antimicrobials (ampicillin, ciprofloxacin, nalidixic acid, sulfisoxazole, streptomycin, and tetracycline), with bar plot validation of their occurrence. Pathogen isolates were further categorized by the number of antimicrobials showing resistance, producing subsets Data_AMR_1 through Data_AMR_6 (e.g., Data_AMR_6 contains isolates resistant to six antimicrobials). For each multiple antimicrobial resistance (M-AMR) scenario, AMR distributions were examined across pathogens, while associated genes were identified (bar plots). Finally, results were compared across M-AMR scenarios (e.g., from Data_AMR_1 to Data_AMR_6) to identify co-resistance patterns and multi-antimicrobial resistance trends. Because PCA and hierarchical clustering are exploratory, unsupervised methods rather than hypothesis-testing procedures, no statistical significance levels (α) were applied in this study.

#### 2.2.1. Principal Component Analysis, Hierarchical Clustering, and Occurrence Bar Plotting

**Figure 2:**
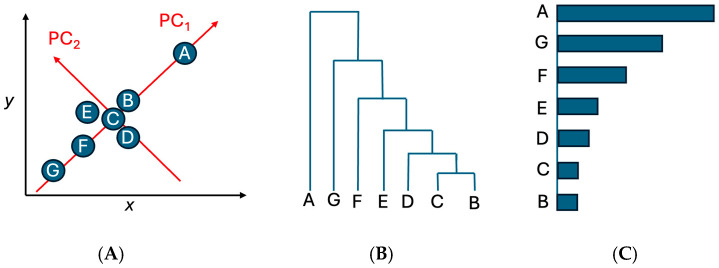
The schematic of the multivariate workflow used in this study. (**A**) PCA projection of Genes A–G for visualization; (**B**) hierarchical clustering of PCA coordinates using Euclidean distance and Ward’s linkage, with the elbow method used to determine cluster boundaries; (**C**) occurrence bar plotting to validate and prioritize genes based on their observed frequencies across isolates.

The analysis began with a gene–antimicrobial matrix in which each row represented an AMR gene and each column represented resistance to a particular antimicrobial. Each matrix entry corresponded to the number of isolates in which the gene co-occurred with resistance to that antimicrobial, enabling quantification of population-level relationships. PCA was performed in R (version 2025.05.1) using *prcomp*(). Projections onto the first two principal components (PC1 and PC2) provided a low-dimensional visualization of variation in co-resistance patterns (Figure 2A), but PCA was not used to define outliers. Instead, PCA served mainly to project high-dimensional profiles into a two-dimensional space suitable for visualization and subsequent clustering. Hierarchical clustering was then applied to the PCA coordinates using Euclidean distance and Ward’s linkage (*hclust*(method = “ward.D2”)) to systematically identify distinct groups of genes. To determine the appropriate number of clusters in an objective and reproducible manner, dendrogram merge heights were evaluated, and an elbow-type criterion was applied (Figure 2B). Genes belonging to clusters that separated clearly from the main aggregated cluster were designated as clustering outliers. These clustering-defined outliers represent genes with distinct co-resistance signatures compared with the bulk distribution. This approach avoids relying on PCA distance alone and ensures that outlier detection is driven by consistent, algorithmic grouping patterns. These clustering-defined outliers were then validated and prioritized using occurrence bar plotting, in which the total number of isolates carrying each gene was quantified using the R command *ggplot2*(). This final validation step ensured that selected genes were not only structurally distinct in clustering space but also frequently observed across isolates, thereby identifying the most epidemiologically meaningful features. Only genes supported by both multivariate structure (clustering) and real-world prevalence (bar plotting) were retained as important contributors to resistance patterns.

#### 2.2.2. Distribution of Antimicrobial Resistance Cases in the U.S.

To characterize how pathogens, antimicrobial combinations, and resistance genes interact across isolates, we generated a series of visualizations in the Section 3 using a flexible R workflow that accommodates resistance to any number of antimicrobials. For each AMR category (AMR_1 through AMR_6), we enumerated all possible antimicrobial combinations using the *combn*() function in R (version 2025.05.1), where the second argument (e.g., 1, 2, …, 6) specifies the size of the combination to compute co-resistance for any *k*-antimicrobial combination. For every antimicrobial combination, we quantified the number of isolates simultaneously resistant to all antimicrobials in that combination. These results were summarized in structured tables and later visualized in the Section 3 as ranked combination tables to highlight the most frequent co-resistance patterns for AMR_1–AMR_6.

To examine three-way relationships among pathogens, antimicrobial combinations, and AMR genes, we constructed a pathogen × antimicrobial-combination × gene array. Each entry in this array represented the number of isolates belonging to a given pathogen, resistant to a specified antimicrobial combination, and carrying a particular AMR gene. Although no figure is included in this section, Section 3 presents bubble-plot–style visualizations derived from this array, where (i) pathogens appear on one axis, (ii) antimicrobial combinations on the other, and (iii) bubble size encodes the number of isolates. Gene labels and label sizes are mapped to their total frequencies across combinations, allowing highly prevalent genes to emerge visually. These visualizations allow us to see, at a glance, how resistance genes concentrate around particular antimicrobial combinations and how these patterns differ across pathogens, thereby providing a concise but powerful representation of the key drivers and interactions shaping antimicrobial resistance profiles in the dataset.

## 3. Results

The top six antimicrobials were first identified from data via multivariate statistical analysis. The resistance patterns were then examined systematically across all AMR scenarios, beginning with isolates resistant to a single antimicrobial (1-AMR) and extending to those resistant to multiple antimicrobials (up to 6-AMR). For each AMR category, we first identified the most frequent antimicrobial combinations and then quantified how these combinations associate with specific pathogens and AMR genes. These relationships were visualized through pathogen–antimicrobial–gene interaction bubble plots, which integrated three dimensions of information: (i) the pathogen contributing to a given resistance event, (ii) the antimicrobial combination defining the resistance category, and (iii) the AMR genes most commonly observed among isolates within that combination. This structure enables direct comparison across AMR levels and highlights how resistance profiles evolve as isolates acquire resistance to additional antimicrobials.

### 3.1. Identification of Top Antimicrobials in the Dataset

**Figure 3:**
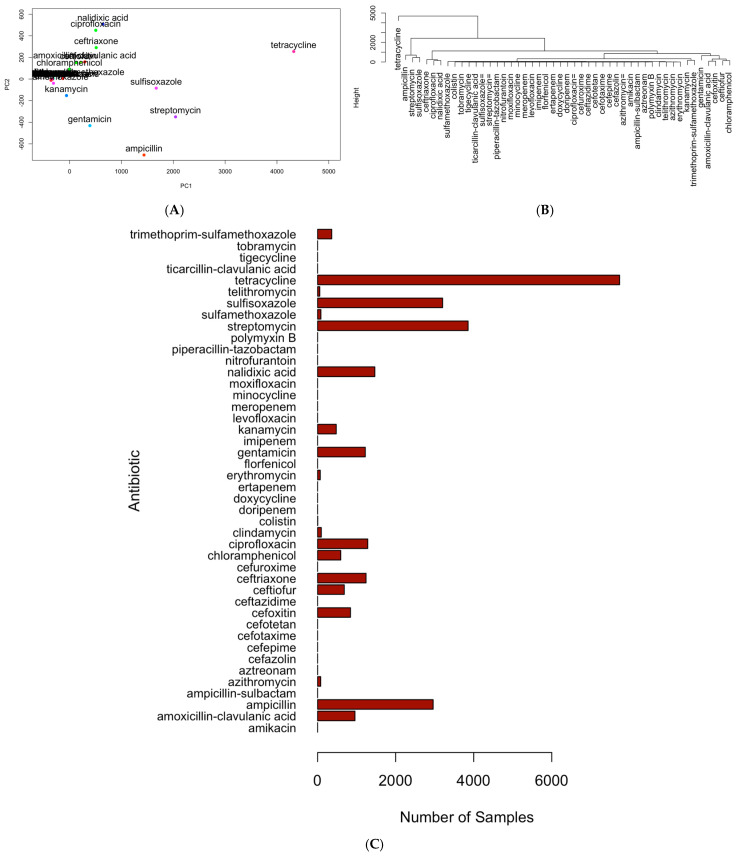
Illustration of the multivariate workflow used to identify representative antimicrobials. (**A**) PCA projection of antimicrobials with detected resistance in foodborne pathogens (2015–2025), showing overlapping clusters of antimicrobials with similar resistance patterns detected across isolates; (**B**) hierarchical clustering of PCA coordinates using Euclidean distance and Ward’s linkage, with cluster boundaries determined by an elbow criterion to separate antimicrobials that were closely grouped in the PCA space; (**C**) bar plot of resistance case counts for each antimicrobial. The overlapping patterns observed in panel (**A**) highlight the limitations of PCA for fully separating antimicrobials, while the dendrogram in panel (**B**) resolves these clusters, and panel (**C**) quantitatively confirms the selection of the top antimicrobials based on resistance frequency.

Figure 3 is used to illustrate the application of principal component analysis (PCA), hierarchical clustering, and bar histograms to identify the top six antimicrobials from the dataset, including tetracycline, ampicillin, streptomycin, sulfisoxazole, nalidixic acid, and ciprofloxacin. In this analysis, PCA was applied to the antimicrobial resistance matrix to project all antimicrobials into a two-dimensional space defined by PC1 and PC2, with 95.3% percentage variance explained by PC1 and PC2 (Figure 3A). This PCA projection served primarily as a visualization tool: antimicrobials with similar resistance profiles tended to cluster together in the PCA space, resulting in several antimicrobials being “lumped” closely along the main axes. Because PCA alone does not cleanly separate antimicrobials, hierarchical clustering was applied to the PCA coordinates using Euclidean distance and Ward’s linkage to systematically distinguish groups of antimicrobials with shared resistance signatures (Figure 3B). The clustering dendrogram was interpreted using an elbow-type criterion, allowing antimicrobials that separated clearly from the bulk clusters to be identified as structurally distinct. These separated branches highlight antimicrobials with resistance patterns that differ substantially from the rest of the dataset. Finally, bar plots (Figure 3C) were generated to quantify the number of resistant isolates for each antimicrobial. This quantitative view validates the selection of the six antimicrobials, including tetracycline, streptomycin, sulfisoxazole, ampicillin, nalidixic acid, and ciprofloxacin, by showing that they also have the highest resistance frequencies, reinforcing the combined conclusions from PCA and clustering. These six antimicrobials additionally span the major antimicrobials represented in the dataset, ensuring that they capture the dominant and clinically relevant resistance phenotypes. As such, they serve as a representative set for downstream multi-antimicrobial resistance analyses.

### 3.2. Resistance to a Single Antimicrobial: Pathogen and Gene Distributions

A total of six antimicrobials, including tetracycline, streptomycin, sulfisoxazole, ampicillin, nalidixic acid, and ciprofloxacin, accounted for all single-drug resistance cases detected in the dataset. As shown in Table 2, tetracycline resistance was the most prevalent (2167 isolates), followed by streptomycin (1452 isolates) and sulfisoxazole (804 isolates). Ampicillin, nalidixic acid, and ciprofloxacin showed lower but non-negligible resistance frequencies. This distribution mirrors the antimicrobial usage patterns and selective pressures commonly reported in food animal production systems, where tetracyclines, aminoglycosides, and sulfonamides have historically been used more extensively, resulting in a larger reservoir of resistant isolates.

**Figure 4:**
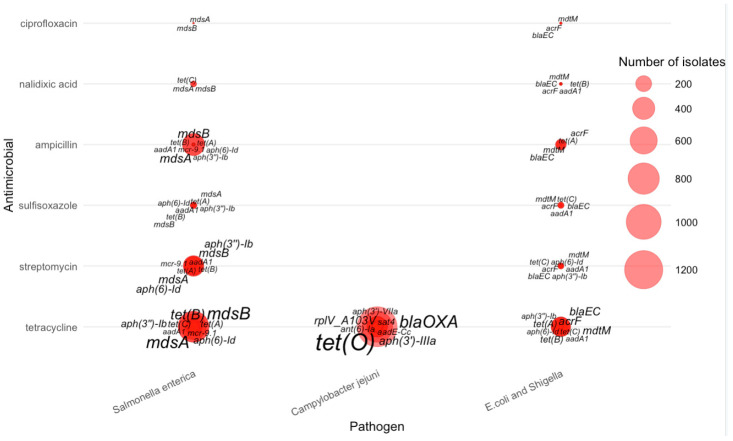
Distribution of AMR genes across pathogens for single antimicrobial resistance cases.

The integrated pathogen–antimicrobial–gene bubble plot further illustrates how single-drug resistance cases are distributed across the three major pathogens (Figure 4). *Salmonella enterica* accounted for the majority of single-AMR events, especially for tetracycline, streptomycin, and ampicillin. *Campylobacter jejuni* dominated resistance to tetracycline. *E. coli*/*Shigella* contributed a smaller but distinct set of resistance cases, particularly for ampicillin and certain tetracycline-associated genes. These pathogen-specific patterns reinforce that single-drug resistance is not uniformly distributed across taxa but is shaped by distinct ecological and selective environments.

The figure further reveals that for tetracycline resistance, the most prominent genes were *tet(O)*—displayed with the largest label size due to its high frequency in *Campylobacter jejuni*—along with *mdsA*, *mdsB*, *aph(6)-Id*, and several other *tet* variants that appeared predominantly in *Salmonella enterica* and *E. coli*/*Shigella*. Streptomycin resistance was strongly associated with aminoglycoside-modifying enzymes, especially *aph(3″)–Ib*, *aph(6)–Id*, *aadA1*, and *mcr-9*, which appeared frequently in *Salmonella* isolates.

For sulfisoxazole, resistance was primarily linked to *mdsA*, *mdsB*, *tet(A)*, and *aph(3″)–Ib*, rather than *sul1* or *sul2*, which do not appear in this dataset. Ampicillin-resistant isolates were marked by β-lactamase genes such as *bla_EC_*, often accompanied by multidrug efflux components such as *mdtM* and *acrF*. Nalidixic acid resistance was comparatively sparse and dominated by *mdsA*, *mdsB*, and *tet(C)*. Ciprofloxacin resistance showed a similar pattern, with *mdsA* and *mdsB* again contributing substantially, suggesting that efflux-mediated resistance is more common than plasmid-borne fluoroquinolone determinants in AMR-1 isolates of these pathogens.

Overall, the AMR-1 bubble map demonstrates pathogen-specific resistance signatures: multidrug efflux genes (*mdsA*/*mdsB*) are broadly distributed across all organisms, *tet(O)* is strongly enriched in *Campylobacter jejuni*, and β-lactamases (*bla* genes) dominate ampicillin resistance in *E. coli*/*Shigella*. These patterns provide a quantitative basis for understanding core resistance determinants associated with single-drug resistance across major foodborne pathogens.

### 3.3. Resistance to Two Antimicrobials: Pathogen and Gene Distributions

Table 3 summarizes co-resistance patterns among isolates resistant to exactly two antimicrobials. Across all observed two-drug combinations, tetracycline appeared most frequently, partnering with streptomycin in 1198 isolates—the dominant co-resistance pair in the dataset. Several other tetracycline-based combinations were also common, including tetracycline–sulfisoxazole (581 isolates) and tetracycline–ampicillin (368 isolates), indicating that tetracycline resistance frequently co-occurs with multiple other drug resistances. Fluoroquinolone co-resistance was also evident: nalidixic acid–ciprofloxacin resistance was the fourth most common combination (244 isolates), reflecting cross-resistance within this drug class. Moderate-frequency pairs included streptomycin–sulfisoxazole (176 isolates) and streptomycin–ampicillin (78 isolates). Less frequent combinations—such as tetracycline with nalidixic acid (13 isolates) or ciprofloxacin (7 isolates)—were also detectable but occurred at much lower rates. Overall, the AMR-2 distribution shows that co-resistance is dominated by a small number of high-frequency antimicrobial pairs, many of which involve tetracycline, highlighting its central role in early multi-drug resistance patterns.

**Figure 5:**
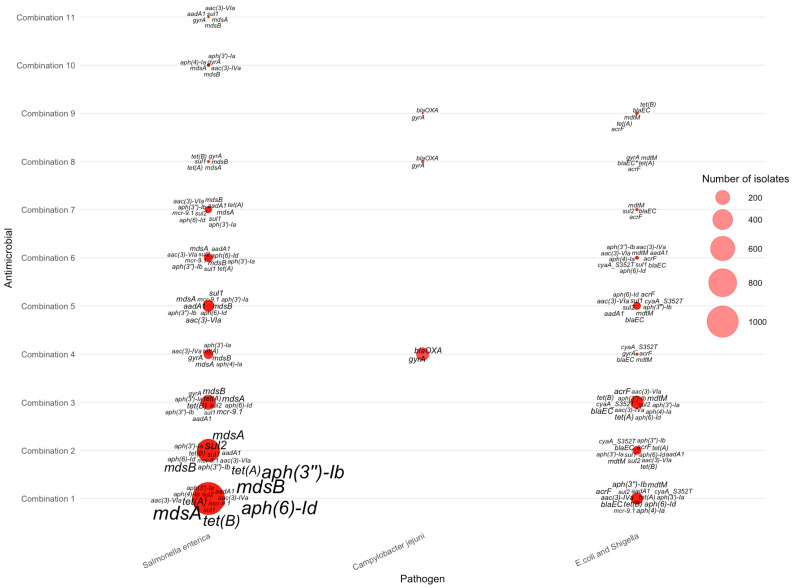
Distribution of AMR genes across pathogens for two-antimicrobial resistance combinations. The antimicrobials for each combination can be found in Table 3.

For isolates resistant to two antimicrobials, distinct pathogen-specific gene signatures emerged across the ranked antimicrobial combinations (Figure 5). *Salmonella enterica* contributed the majority of AMR-2 isolates and displayed the broadest repertoire of resistance genes. Across the highest-frequency combinations—particularly tetracycline–streptomycin, tetracycline–sulfisoxazole, and tetracycline–ampicillin—*Salmonella* exhibited consistent enrichment of *mdsA*, *mdsB*, *aph(6)-Id*, *aph(3″)-Ib, tet(A)*, and *tet(B)*, with *mdsA/mdsB* and *aph(6)-Id* appearing as the most dominant and widespread determinants. In contrast, *Campylobacter jejuni* showed a much narrower gene profile, centered primarily on *bla_OXA_*, and *gyrA*, which clustered mainly around nalidixic acid–ciprofloxacin co-resistance. *E. coli*/*Shigella* isolates displayed intermediate diversity, frequently carrying *bla_EC_*, *mdtM*, *acrF*, and *aph(3′)-Ib*, with enriched signatures observed in combinations involving ciprofloxacin and nalidixic acid. Collectively, the AMR-2 bubble plot highlights how the transition from single-drug to dual-drug resistance reveals clearer pathogen-specific gene groupings: *Salmonella* retains a wide multidrug-efflux– and aminoglycoside-associated signature, *Campylobacter* shows quinolone-focused markers, and *E. coli*/*Shigella* carry a mix of β-lactam, efflux, and aminoglycoside resistance genes.

### 3.4. Resistance to Three Antimicrobials: Pathogen and Gene Distributions

Table 4 summarizes the distribution of isolates resistant to three antimicrobials (AMR-3), revealing several dominant three-drug resistance patterns among foodborne pathogens. The most frequent combinations—tetracycline + nalidixic acid + ciprofloxacin (504 isolates), tetracycline + streptomycin + sulfisoxazole (494 isolates), tetracycline + sulfisoxazole + ampicillin (474 isolates), and tetracycline + streptomycin + ampicillin (407 isolates)—all involve tetracycline as a shared component, underscoring its central role in multidrug resistance. Streptomycin, sulfisoxazole, and ampicillin also appear repeatedly across the highest-frequency combinations, suggesting strong co-selection among these antimicrobials within circulating strains. In contrast, lower-frequency combinations (fewer than 10 isolates) such as tetracycline + streptomycin + ciprofloxacin or streptomycin + nalidixic acid + ciprofloxacin occur only sporadically, indicating that most triple-resistant isolates cluster into a small number of recurrent resistance profiles.

**Figure 6:**
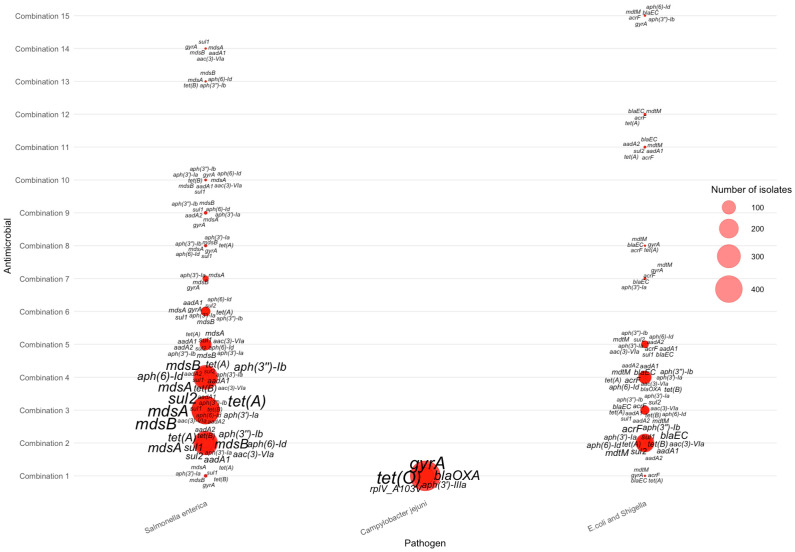
Distribution of AMR genes across pathogens for three-antimicrobial resistance combinations. The antimicrobials for each combination can be found in Table 4.

For isolates resistant to three antimicrobials, gene–pathogen patterns varied substantially across the top combinations listed in Table 4. The most common AMR-3 profiles—tetracycline–nalidixic acid–ciprofloxacin (Combination 1), tetracycline–streptomycin–sulfisoxazole (Combination 2), tetracycline–sulfisoxazole–ampicillin (Combination 3), and tetracycline–streptomycin–ampicillin (Combination 4)—were dominated by *Salmonella enterica*, which exhibited large clusters of multidrug-resistance genes (Figure 6). Across these combinations, *Salmonella enterica* consistently carried *mdsA*, *mdsB*, *tet(A)*, *tet(B)*, *sul2*, *aph(3″)-Ib*, and *aph(6)-Id*, forming the core signature of triple-antimicrobial resistance in these species. In contrast, *Campylobacter jejuni* contributed fewer AMR-3 cases but displayed distinct markers, most notably *tet(O)*, *gyrA*, and *bla_OXA_*, consistent with known tetracycline and fluoroquinolone resistance mechanisms. *E. coli* and *Shigella* showed more heterogeneous genetic profiles, frequently expressing *bla_EC_*, *acrF*, *aadA1*, and *mdtM* across lower-frequency combinations. These patterns collectively demonstrate that high-frequency AMR-3 resistance is primarily driven by *Salmonella* through efflux pump and aminoglycoside-associated genes, whereas *Campylobacter* and *E. coli*/*Shigella* contribute pathogen-specific resistance determinants linked to fluoroquinolone and β-lactam resistance.

### 3.5. Resistance to Four Antimicrobials: Pathogen and Gene Distributions

Table 5 summarizes the distribution of isolates resistant to four antimicrobials (4-AMR), revealing a highly skewed pattern dominated by a single tetracycline-centered profile. The most frequent 4-AMR combination—tetracycline + streptomycin + sulfisoxazole + ampicillin—accounted for 649 isolates, far exceeding all other four-drug combinations and reflecting a consistent co-resistance pattern among these long-used antimicrobials in foodborne pathogens. The next most common combinations occurred at substantially lower frequencies, such as tetracycline + sulfisoxazole + nalidixic acid + ciprofloxacin (84 isolates) and tetracycline + sulfisoxazole + ampicillin + nalidixic acid (39 isolates), indicating that multi-class resistance involving fluoroquinolones or quinolones occurs but is less widespread. Several additional 4-AMR combinations were detected at very low levels (≤10 isolates), suggesting sporadic or lineage-specific resistance events.

**Figure 7:**
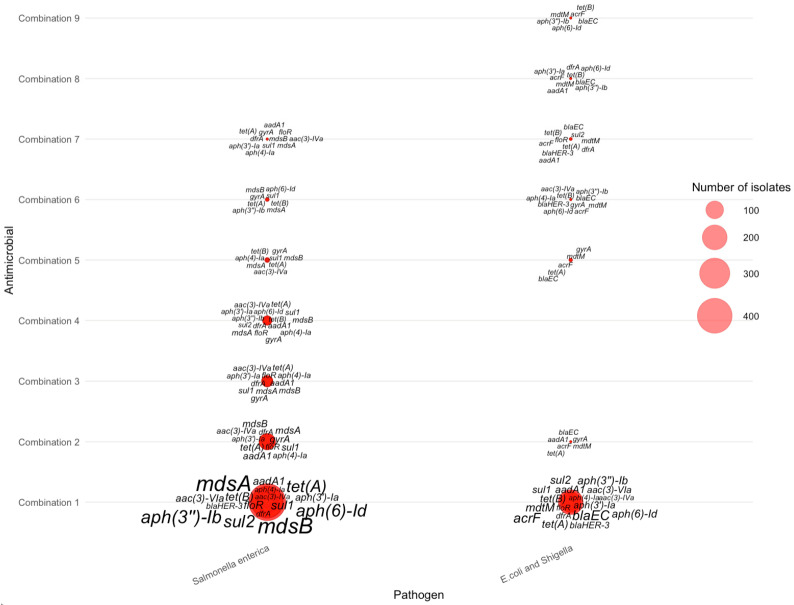
Distribution of AMR genes across pathogens for four-antimicrobial resistance combinations. The antimicrobials for each combination can be found in Table 5.

Across the nine AMR-4 antimicrobial combinations (Table 5), resistance gene distributions were concentrated almost entirely in *Salmonella enterica* and *E. coli*/*Shigella*, with no AMR-4 isolates detected for *Campylobacter jejuni*. In *Salmonella enterica*, combinations 1–4—which included tetracycline, streptomycin, sulfisoxazole, and ampicillin—showed the highest gene burdens, characterized by frequent multidrug-resistance loci such as *mdsA*, *mdsB*, *aph(3″)-Ib*, *aph(6)-Id*, *sul2*, and *tet(A)*. These genes appeared in large clusters (as shown in Figure 7), reflecting the strong co-selection pressures associated with these four drugs in *Salmonella enterica*. In contrast, *E. coli*/*Shigella* displayed smaller AMR-4 gene clusters, typically involving combinations containing nalidixic acid or ciprofloxacin. Key genes in these isolates included *bla_EC_*, *acrF*, *mdtM*, *gyrA*, and *tet(B)*, which together indicate quinolone and β-lactam resistance signatures commonly seen in Enterobacterales. The sharp contrast between the dense *Salmonella enterica* clusters and the sparse *E. coli*/*Shigella* clusters illustrates the species-specific structure of AMR-4 profiles and highlights *Salmonella enterica* as the dominant contributor to high-order multi-antimicrobial resistance.

### 3.6. Resistance to Five Antimicrobials: Pathogen and Gene Distributions

Table 6 summarizes the distribution of isolates exhibiting resistance to five antimicrobials. Similar to lower-order resistance patterns, tetracycline, streptomycin, and sulfisoxazole again form the backbone of the most common multidrug resistance profiles. The most frequent AMR-5 combination—tetracycline + streptomycin + sulfisoxazole + nalidixic acid + ciprofloxacin—accounted for 153 isolates, indicating that this core set of antimicrobials represents the dominant multidrug resistance signature in the dataset. The second most common combination—tetracycline + sulfisoxazole + ampicillin + nalidixic acid + ciprofloxacin—included 76 isolates, followed by tetracycline + streptomycin + sulfisoxazole + ampicillin + nalidixic acid with 30 isolates. All remaining AMR-5 combinations occurred at much lower frequencies (≤4 isolates each), highlighting the sharp drop-off in diversity as resistance expands to five antimicrobials. Collectively, these findings demonstrate that although a wide range of theoretical five-drug combinations is possible, only a small number appear frequently in circulating foodborne pathogens, and they consistently involve tetracycline, streptomycin, and sulfisoxazole—reinforcing their central role in multidrug resistance profiles.

**Figure 8:**
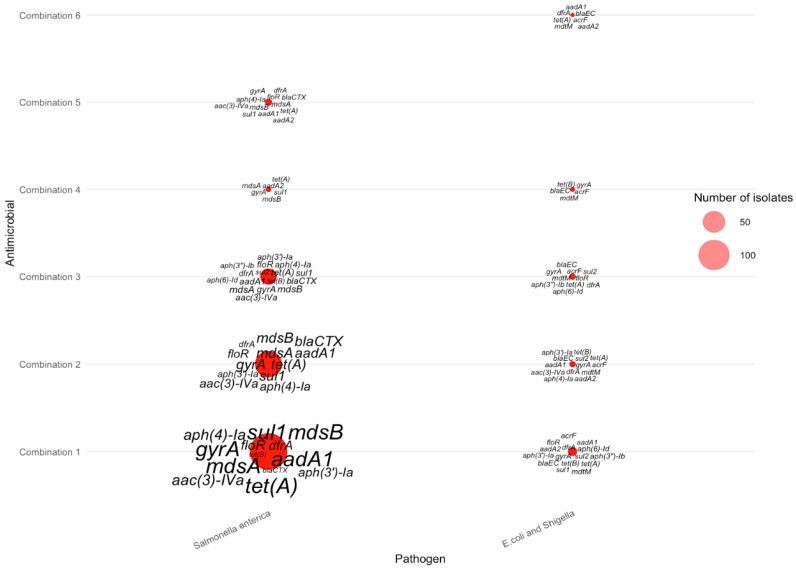
Distribution of AMR genes across pathogens for five-antimicrobial resistance combinations. The antimicrobials for each combination can be found in Table 6.

For isolates resistant to five antimicrobials, the six combinations listed in Table 6 produced distinct pathogen–gene patterns, as shown in the corresponding bubble plot. Most AMR-5 isolates belonged to *Salmonella enterica*, with Combination 1 (tetracycline–streptomycin–sulfisoxazole–nalidixic acid–ciprofloxacin; 153 isolates) contributing the largest cluster. Within this group, several multidrug-efflux and aminoglycoside-modifying genes—including *mdsA*, *mdsB*, *gyrA*, *tet(A)*, *floR*, *aadA1*, and *aph(3″)-Ia*,—were highly enriched (as shown in Figure 8), reflecting broad-spectrum resistance mechanisms. Combination 2 (tetracycline–sulfisoxazole–ampicillin–nalidixic acid–ciprofloxacin; 76 isolates) showed a similar genetic profile, again dominated by *mdsA*, *mdsB*, *aac(3)-IVa*, *tet(A)*, and *aadA1*, with slightly fewer total occurrences. Combination 3 (tetracycline–streptomycin–sulfisoxazole–ampicillin–nalidixic acid; 30 isolates) displayed a reduced but consistent pattern, primarily featuring *mdsA*, *mdsB*, *tet(A)*, and *aph(3″)-Ib*.

In contrast, *E. coli* and *Shigella* isolates showed far fewer AMR-5 cases, producing smaller and more dispersed bubbles across combinations. The most recurrent genes among these isolates included *bla_EC_*, *mdtM*, *acrF*, *tet(B)*, and *aac(3″)-Ia*. As in previous AMR scenarios, *Campylobacter jejuni* contributed no AMR-5 cases, consistent with the absence of bubbles in the corresponding positions of the visualization. Together, these distributions highlight that five-antimicrobial resistance remains concentrated in *Salmonella enterica*, driven by a recurring set of efflux pumps, aminoglycoside-modifying enzymes, and tetracycline resistance determinants.

### 3.7. Resistance to Six Antimicrobials: Pathogen and Gene Distributions

Since all six antimicrobials are included, only one combination exists. In total, there are 164 cases that were taken from the NCBI database. These recorded cases only represent a much smaller percentage than those cases that have AMR to fewer than six antimicrobials.

**Figure 9:**
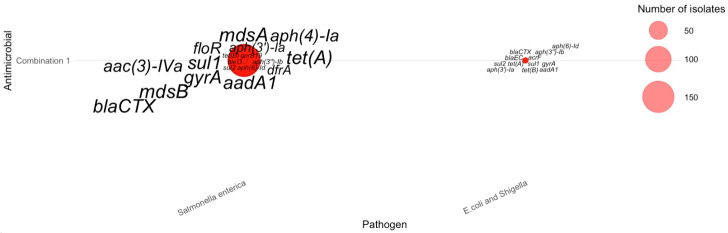
Distribution of AMR genes across pathogens for six-antimicrobial resistance combinations. The antimicrobials for each combination can be found in Table 2.

For the AMR-6 scenario, a single antimicrobial combination—tetracycline, streptomycin, sulfisoxazole, nalidixic acid, ampicillin, and ciprofloxacin had isolates resistant to all six drugs. The gene-level bubble plot reveals that these highly multidrug-resistant isolates were detected exclusively in *Salmonella enterica* and *E. coli*/*Shigella*, with no AMR-6 cases observed in *Campylobacter jejuni* (as shown in Figure 9). In *Salmonella enterica*, the dominant resistance determinants included *mdsA*, *mdsB*, *aph(4′)-la*, *aac(3)-IVa*, *sul1*, *aadA1*, *floR*, *gyrA*, *tet(A)*, and *bla_CTX_*, illustrating a broad suite of efflux pump, aminoglycoside-modifying, sulfonamide, tetracycline, and β-lactam resistance genes co-occurring within the same isolates. In contrast, *E. coli*/*Shigella* isolates resistant to all six antimicrobials carried a smaller yet distinct combination of genes—most prominently *bla_EC_*, *bla_CTX_*, *acrF*, *tet(A)*, *tet(B)*, *aph(3′)-Ib*, *aph(6)-Id*, and *aadA1*. These patterns demonstrate that although AMR-6 cases are rare, the few isolates exhibiting this extreme phenotype harbor dense clusters of resistance determinants, with *Salmonella enterica* showing a broader and more diverse gene repertoire compared with *E. coli*/*Shigella*.

### 3.8. Comparative Analysis Across All 6 AMR Scenarios

Table 7 summarizes the distribution of pathogen isolates across the six AMR categories using row-wise percentages, ensuring that each row sums to 100%. This structure reveals clear species-specific resistance patterns. *Campylobacter jejuni* appears overwhelmingly in the 1-AMR category, comprising 65.7% of all isolates resistant to only one antimicrobial, and is nearly absent in higher AMR categories, confirming that multidrug resistance is extremely rare in this species. In contrast, *Salmonella enterica* shows a broader distribution across AMR levels: although it represents 19.5% of 1-AMR isolates, its highest contribution occurs in 2-AMR (35.7%), followed by gradual declines through 3-AMR (20.4%), 4-AMR (10.3%), 5-AMR (4.2%), and 6-AMR (2.7%). *E. coli* and *Shigella* display a more even distribution across 1-AMR to 4-AMR, with smaller contributions in the 5-AMR and 6-AMR groups. The antimicrobial rows show a different trend: tetracycline, sulfisoxazole, streptomycin, ampicillin, nalidixic acid, and ciprofloxacin become nearly universal within the higher AMR categories—approaching or reaching 100% representation in 4-AMR through 6-AMR—highlighting their central role in multi-antimicrobial resistance patterns. Overall, this table clarifies how pathogen species differ sharply in their AMR complexity, while core antimicrobials consistently dominate higher-order resistance combinations.

## 4. Discussion

### 4.1. Antimicrobials and Pathogens Involved in Microbial Resistance in Food Animals

Across all resistance categories (AMR-1 through AMR-6), a consistent and biologically meaningful hierarchy of antimicrobials emerged from our analysis. Tetracycline > streptomycin > sulfisoxazole > ampicillin > nalidixic acid > ciprofloxacin were repeatedly dominant across single- and multi-drug resistance categories, and they constituted nearly all of the highest-frequency antimicrobial combinations observed in 2-AMR through 6-AMR scenarios. Their prominence reflects both extensive historical use in agricultural and clinical settings and well-documented selective pressures that facilitate persistence of associated resistance genes. Tetracycline resistance was nearly ubiquitous across higher-order AMR categories, reaching 94–100% representation in 3-AMR to 6-AMR isolates. This confirms tetracycline’s role as a central driver of multi-antimicrobial resistance, consistent with global findings that tetracycline resistance genes (*tetA*, *tetB*, *tetO*) are among the most widespread determinants in Enterobacteriaceae and foodborne pathogens [9]. Sulfisoxazole, streptomycin, and ampicillin also increased sharply in prevalence as the number of co-resistant antimicrobials grew, indicating that these agents form the backbone of the most common co-resistance clusters circulating in *Salmonella enterica* and *E. coli*/*Shigella*. In contrast, nalidixic acid and ciprofloxacin—representing fluoroquinolone resistance—were less common in low-order AMR but became strongly enriched in the highest AMR levels, a pattern consistent with the evolutionary acquisition of gyrase- and efflux-mediated mechanisms at later stages of multidrug resistance [36]. Collectively, these findings suggest that the six highlighted antimicrobials capture the major phenotypic “axes” of resistance in U.S. foodborne pathogens, enabling a representative and interpretable framework for multivariate visualization and cross-scenario comparisons.

The three foodborne pathogens differ markedly in their contributions across resistance categories. *Campylobacter jejuni* overwhelmingly dominates AMR-1 (65.7%) but rarely appears in higher resistance categories, being absent entirely from AMR-4 through AMR-6. This pattern aligns with *Campylobacter*’s known dominance in single-drug resistance (especially tetracycline and fluoroquinolones) but low association with many-drug resistance. In contrast, *Salmonella enterica* is broadly distributed across all AMR levels—from 19.5% of AMR-1 cases up to 61% of AMR-4 and 94%+ in AMR-5 and AMR-6—making it the primary driver of complex multidrug resistance [37]. *E. coli*/*Shigella* contributes modestly to low-level resistance but retains representation up to AMR-6, especially in fluoroquinolone-associated multidrug cases. The bubble-plot visualizations reinforce these trends: *Salmonella enterica* dominates the gene clouds in nearly every AMR-k figure, while *E. coli*/*Shigella* contributes smaller but consistent clusters.

### 4.2. AMR Gene Landscape

Across all resistance scenarios, several antimicrobial resistance (AMR) genes emerged as dominant contributors to the observed patterns. Most notably, *mdsA* and *mdsB* were consistently the most frequently present genes in resistant isolates. Their prevalence highlights the widespread nature of the *mds* efflux pump system, even though these genes do not directly encode resistance to the six antimicrobials studied [38,39]. Interestingly, nearly every isolate containing *mdsA* also contained *mdsB*. The co-dominance of these genes indicates that efflux-pump mechanisms are central to the broader multi-antimicrobial resistance phenotype, rather than being tied to a single antimicrobial class. Their abundance also reflects the predominance of *Salmonella enterica* in the dataset, since this pathogen frequently carries *mds* genes. The tetracycline-resistance genes *tet(A)* and *tet(B)* were also highly prevalent, complementing the widespread phenotypic tetracycline resistance observed across AMR categories [2].

Streptomycin resistance genes were another recurring feature, with aminoglycoside-modifying enzyme (AME) genes—such as *aph(3″)-Ib*, *aph(6)-Id*, and *aadA1*—appearing prominently in all six resistance categories. These genes encode enzymes that modify aminoglycosides, preventing antimicrobial binding and thereby disabling their antimicrobial activity [40,41]. Sulfonamide resistance genes *sul1* and *sul2* also highly detected. These genes encode dihydropteroate synthase (DHPS) variants with reduced affinity for sulfonamides, preventing sulfisoxazole from binding and rendering it ineffective [42]. Although absent in *C. jejuni* isolates (Figure 7), *sul1* and *sul2* were common in *Salmonella enterica*, and *E. coli and Shigella* spp. Their co-occurrence varied by resistance level, with fewer isolates carrying both genes as the number of resistance determinants increased. This may suggest redundancy when multiple resistance mechanisms accumulate.

Quinolone resistance genes (*acrF*, *gyrA*, and *mdtM*) were also widespread across resistance categories. *acrF* and *mdtM* encode efflux pumps that expel quinolones, while *gyrA* mutations prevent antimicrobial binding to the DNA gyrase target site [43,44]. These genes were frequently observed in all three pathogens. Finally, *bla_EC_*, *bla_OXA_*, and *bla_CTX_* encode β-lactamases that hydrolyze the β-lactam ring of ampicillin, preventing effective binding and resulting in resistance [45,46]. They were most common in *E. coli and Shigella*, and *C. jejuni* isolates, but less frequent in *Salmonella enterica*. This is notable because *Salmonella enterica* is historically associated with ampicillin resistance, likely due to other β-lactamases such as *bla_TEM-1_*, which were not included in this dataset.

### 4.3. Interaction of Genes, Pathogens, and Antimicrobials Across AMR Categories

The interactions among pathogens, antimicrobials, and resistance genes display a clear escalation in complexity from AMR-1 through AMR-6, revealing how co-resistance builds through the coordinated accumulation of specific genetic determinants. In the AMR-1 category, gene–drug relationships are highly antimicrobial-specific: *tet(A)*, *tet(B)*, *mdsA*, and *mdsB* dominate tetracycline resistance in *Salmonella enterica* [47], whereas *tet(O)* and *bla_OXA_* are characteristic of fluoroquinolone-related single-drug resistance in *Campylobacter jejuni* [48]. As co-resistance expands into AMR-2, frequent two-drug combinations—particularly tetracycline plus streptomycin—consistently enrich for *mdsA*, *mdsB*, *tet(A*/*B)*, *aph(3″)-Ib*, and *aph(6)-Id*, indicating that efflux pump genes and aminoglycoside-modifying enzymes form some of the earliest and most stable genetic modules supporting multidrug resistance.

In AMR-3 and AMR-4, the gene profiles broaden substantially, with large “gene clouds” reflecting stacked resistance determinants across multiple antimicrobials. The most common combinations, such as tetracycline–sulfisoxazole–ciprofloxacin and tetracycline–streptomycin–ampicillin, are highly enriched with *sul1*, *sul2*, *tet(A*/*B)*, *mdsA*/*B*, *bla_CTX_*, *aadA1*, *aac(3)-IVa*, and fluoroquinolone-associated *gyrA* mutations. These patterns illustrate convergence between efflux systems, plasmid-borne β-lactamases, aminoglycoside resistance genes, and sulfonamide resistance pathways, suggesting coordinated selection pressures favoring multi-class resistance cassettes.

At the highest resistance levels (AMR-5 and AMR-6), the gene landscapes are dominated almost exclusively by *Salmonella enterica*, which carries dense clusters of resistance determinants. These include efflux pump genes (*mdsA*, *mdsB*, *acrF*, *mdtM*), β-lactamases (*bla_EC_*, *bla_CTX_*), aminoglycoside-modifying enzymes (*aph(3″)-Ib*, *aph(6)-Id*, *aac(3)-IVa*), and chromosomal fluoroquinolone mutations (*gyrA*). These profiles represent highly adapted, extensively resistant *Salmonella enterica* lineages capable of withstanding five or more antimicrobial agents.

Overall, the progression from AMR-1 to AMR-6 demonstrates a stepwise accumulation of resistance: AMR-1 reflects pathogen–drug pairings shaped by intrinsic ecology (*Campylobacter* for tetracycline and fluoroquinolones; *Salmonella enterica* across multiple classes). AMR-2 to AMR-4 mark the emergence of core resistance gene modules involving efflux, tetracycline, sulfonamide, β-lactam, and aminoglycoside pathways. AMR-5 and AMR-6 are characterized by *Salmonella enterica* lineages carrying large mobile cassettes combined with chromosomal mutations, enabling broad-spectrum resistance. These findings highlight the importance of monitoring interactions among these six key antimicrobials and the resistance genes they select for, as well as prioritizing targeted interventions for *Salmonella enterica*—the primary driver of high-level multi-class antimicrobial resistance in U.S. foodborne pathogens.

### 4.4. Limitations and Future Work

There were a couple of key limitations throughout the study that should be noted. Firstly, although the NCBI Pathogen Isolates Browser allowed for a large and informative data set, it was heavily dominated by certain variables like *Salmonella enterica* isolates in terms of pathogens. Therefore, the distribution of the different isolates was densely packed into specific categories. This could affect the trends towards what is most predictable for *Salmonella enterica*. At the same time, the sheer abundance of *Salmonella enterica* isolates highlights the pathogen’s prominence or, equivalently, the importance of surveilling its trends. Additionally, these isolates were only sourced from the U.S., limiting the scope of understanding of AMR. Due to the limited space and the large number of potential combinations, we only analyzed the top 6 antimicrobials and top 3 pathogens, which may mean potential trends and patterns were disregarded as a result of a narrow focus. In addition, many of the top 20 genes could correlate with some other important antimicrobials that were not among the top 6, leading to some results that may not be generalizable. Another substantial limitation was that *Campylobacter jejuni* had no cases of resistance against four, five, and six antimicrobials. This pathogen was still resisted by antimicrobials other than the six selected ones. Lastly, another key limitation of this study is the lack of consistent, fine-grained information on the specific source context of isolates within each food-animal host category. Although isolates are annotated by host species (chicken, turkey, beef, and pork), the NCBI Pathogen Isolates Browser does not uniformly specify whether individual isolates originated from production environments, processing facilities, retail meat products, or foodborne outbreak investigations involving human cases. As a result, no additional filtering or stratification by production, processing, retail, or outbreak source was applied beyond the standardized host annotations provided by NCBI. Consequently, the resistance patterns reported here should be interpreted as aggregated, host-associated AMR profiles rather than source-specific risk estimates.

In future work, more types of pathogens along with more types of antimicrobials could potentially be investigated, especially for the broader patterns between them. A broader range of pathogens and antimicrobials would also be useful to have a better understanding of the extent of multi-antimicrobial resistance these pathogens possess. In addition, incorporating datasets with more detailed and standardized isolate source annotations could enable stratified analyses that distinguish among on-farm production, processing, retail, and human outbreak–associated contexts, thereby further enhancing the interpretability and translational relevance of antimicrobial resistance surveillance data. Additionally, the dataset could have been expanded to be larger (i.e., more samples of each pathogen) to achieve an even greater understanding of the patterns between AMR and the phenotype, despite this study already conducting research on quite a large dataset. Expanding the sourcing of the data internationally could also be useful to identify whether trends in the U.S. align with trends worldwide. Additionally, while foodborne pathogens are very dangerous, there are other equally, if not more dangerous, pathogens sourced from humans that could also be investigated for patterns between the genome and resistance behaviors. With an understanding of the resistance genome and its connection to resistance behavior in pathogens, future work could potentially include modeling the spread of such pathogens and incorporating the potential severity for certain organisms.

## 5. Conclusions

This study presents a unified framework for understanding the evolution of antimicrobial resistance (AMR) in foodborne pathogens by integrating antimicrobial, pathogen, and gene-level patterns across resistance levels from AMR-1 to AMR-6. Resistance emerges through a coordinated, stepwise process, progressing from antimicrobial-specific gene associations to densely layered resistance gene modules at higher AMR levels. Across all categories, six antimicrobials—tetracycline, streptomycin, sulfisoxazole, ampicillin, nalidixic acid, and ciprofloxacin—consistently define the dominant axes of co-resistance, reflecting long-standing selective pressures in food-animal production systems. A clear species stratification is observed: *Campylobacter jejuni* is largely confined to single-drug resistance, whereas *Salmonella enterica* drives nearly all high-order multidrug resistance (AMR-5 and AMR-6) through the accumulation of efflux, aminoglycoside-modifying, sulfonamide, β-lactamase, and fluoroquinolone-associated genes. Together, these findings demonstrate that multidrug resistance arises through the progressive assembly of stable resistance gene modules rather than random events, underscoring the importance of integrated phenotypic and genotypic surveillance to anticipate AMR progression in U.S. food-animal systems.

## Figures and Tables

**Table 1:** Structure and illustrative subset of metadata, antimicrobial susceptibility, and AMR gene profiles in poultry isolates.

Organism	Host/Source	Location	Collection Date	Ampicillin	…	Cefazolin	…	Gene_1	…	Gene_163
1	1	33	2015	1	…	1	…	1	…	0
2	2	23	2020	1	…	1	…	0	…	0
3	3	20	2019	0	…	1	…	1	…	1
2	4	4	2018	0	…	1	…	1	…	0
3	1	36	2025	0	…	0	…	0	…	1

Note: Organisms are indexed numerically, with three distinct values (1–3) representing the three pathogens included in this study. Similarly, host animal/source categories are coded as four distinct values (1–4), each corresponding to one of the four food animals sampled (i.e., pigs, cattle, chickens, and turkeys). “…” indicates additional items (i.e., columns) that are omitted due to space constraints.

**Table 2:** The distribution of single AMR cases for pathogens with resistance against one antimicrobial.

Antimicrobial Resistance Isolates Resistant to One Antimicrobial	
Antimicrobial	Count
tetracycline	2167
streptomycin	1427
sulfonazole	804
ampicillin	497
nalidixic acid	265
ciprofloxacin	251

**Table 3:** Numbers of pathogens with detected resistance to specific pairs of antimicrobials.

Antimicrobial Resistance Isolates Resistant to Two Antimicrobials			
Combinations	Antimicrobial 1	Antimicrobial 2	Count
Combination 1	tetracycline	streptomycin	1198
Combination 2	tetracycline	sulfonazole	581
Combination 3	tetracycline	ampicillin	368
Combination 4	nalidixic acid	ciprofloxacin	244
Combination 5	streptomycin	sulfonazole	176
Combination 6	streptomycin	ampicillin	78
Combination 7	sulfonazole	ampicillin	45
Combination 8	tetracycline	nalidixic acid	13
Combination 9	tetracycline	ciprofloxacin	7
Combination 10	ampicillin	nalidixic acid	6
Combination 11	sulfonazole	nalidixic acid	2

**Table 4:** Count of isolates resistant to three antimicrobials.

Antimicrobial Resistance Isolates Resistant to Three Antimicrobials				
Combinations	Antimicrobial 1	Antimicrobial 2	Antimicrobial 3	Count
Combination 1	tetracycline	nalidixic acid	ciprofloxacin	504
Combination 2	tetracycline	streptomycin	sulfonazole	494
Combination 3	tetracycline	sulfonazole	ampicillin	474
Combination 4	tetracycline	streptomycin	ampicillin	407
Combination 5	streptomycin	sulfonazole	ampicillin	95
Combination 6	tetracycline	nalidixic acid	nalidixic acid	42
Combination 7	ampicillin	nalidixic acid	ciprofloxacin	18
Combination 8	tetracycline	ampicillin	nalidixic acid	4
Combination 9	streptomycin	ampicillin	nalidixic acid	4
Combination 10	tetracycline	streptomycin	nalidixic acid	2
Combination 11	tetracycline	sulfonazole	ciprofloxacin	2
Combination 12	tetracycline	ampicillin	ciprofloxacin	2
Combination 13	tetracycline	streptomycin	ciprofloxacin	1
Combination 14	streptomycin	sulfonazole	nalidixic acid	1
Combination 15	streptomycin	nalidixic acid	ciprofloxacin	1

**Table 5:** Count of isolates resistant to four antimicrobials.

Antimicrobial Resistance Isolates Resistant to Four Antimicrobials					
Combinations	Antimicrobial 1	Antimicrobial 2	Antimicrobial 3	Antimicrobial 4	Count
Combination 1	tetracycline	streptomycin	sulfonazole	ampicillin	649
Combination 2	tetracycline	sulfonazole	nalidixic acid	ciprofloxacin	84
Combination 3	tetracycline	sulfonazole	ampicillin	nalidixic acid	39
Combination 4	tetracycline	streptomycin	sulfonazole	nalidixic acid	27
Combination 5	tetracycline	ampicillin	nalidixic acid	ciprofloxacin	10
Combination 6	tetracycline	streptomycin	ampicillin	nalidixic acid	6
Combination 7	tetracycline	sulfonazole	ampicillin	ciprofloxacin	3
Combination 8	tetracycline	streptomycin	sulfonazole	ciprofloxacin	1
Combination 9	tetracycline	streptomycin	ampicillin	ciprofloxacin	1

**Table 6:** Count of isolates resistant to five antimicrobials.

Antimicrobial Resistance Isolates Resistant to Five Antimicrobials						
Combinations	Antimicrobial 1	Antimicrobial 2	Antimicrobial 3	Antimicrobial 4	Antimicrobial 5	Count
Combination 1	tetracycline	streptomycin	sulfonazole	nalidixic acid	ciprofloxacin	153
Combination 2	tetracycline	sulfonazole	ampicillin	nalidixic acid	ciprofloxacin	76
Combination 3	tetracycline	streptomycin	sulfonazole	ampicillin	nalidixic acid	30
Combination 4	tetracycline	streptomycin	ampicillin	nalidixic acid	ciprofloxacin	4
Combination 5	streptomycin	sulfonazole	ampicillin	nalidixic acid	ciprofloxacin	4
Combination 6	tetracycline	streptomycin	sulfonazole	ampicillin	ciprofloxacin	1

**Table 7:** Distribution of isolates resistant to one through six antimicrobials (1–6 AMR) across animal hosts, bacterial species, and antimicrobial agents in the United States (2015–2025).

	Category	1-AMR	2-AMR	3-AMR	4-AMR	5-AMR	6-AMR
Bacteria	*Salmonella enterica*	1157 (19.5%)	2121 (35.7%)	1210 (20.4%)	611 (10.3%)	252 (4.2%)	160 (2.7%)
	*Campylobacter jejuni*	1311 (65.7%)	169 (8.5%)	499 (25.0%)	0 (0.0%)	0 (0.0%)	0 (0.0%)
	*E. coli* and *Shigella*	451 (31.0%)	428 (29.4%)	342 (23.5%)	208 (14.3%)	16 (1.1%)	4 (0.3%)
	Total	2919	2718	2051	819	268	164

## Data Availability

The data supporting the findings of this study are available from the corresponding author upon request.
